# Efficacy of repeated external cueing training on freezing of gait and gait performance in Parkinson’s disease: a systematic review and meta-analysis

**DOI:** 10.3389/fnagi.2026.1808824

**Published:** 2026-05-13

**Authors:** Qingling Sun, Wangfang Yu, Huafu Ni

**Affiliations:** 1 Department of Pharmacy, Beilun People’s Hospital, Ningbo, Zhejiang, China; 2 Department of Neurosurgery, Beilun People’s Hospital, Ningbo, Zhejiang, China; 3 Department of Neurology, Beilun People’s Hospital, Ningbo, Zhejiang, China

**Keywords:** cueing, freezing of gait, meta-analysis, Parkinson’s disease, systematic review

## Abstract

**Background:**

Freezing of gait (FOG) is a disabling gait disturbance in Parkinson’s disease (PD) that responds poorly to pharmacological treatment. External cueing has been proposed as a non-pharmacological strategy to improve gait and mobility; however, the effectiveness of repeated external cueing training on FOG-related outcomes remains uncertain, particularly across different cueing modalities.

**Methods:**

We conducted a systematic review and meta-analysis of randomized controlled trials (RCTs), with a specific focus on repeated external cueing interventions (≥2 weeks). PubMed, Web of Science, Embase, the Cochrane Library, CNKI, Wanfang, and VIP databases were searched from inception to November 10, 2025. RCTs evaluating external cueing interventions in individuals with PD were included. Primary outcomes were FOG severity assessed by the Freezing of Gait Questionnaire (FOGQ) or New FOGQ (NFOGQ). Secondary outcomes included Timed Up and Go (TUG), MDS–UPDRS part III, and Berg Balance Scale (BBS). Risk of bias was assessed using ROB 2.0, and evidence certainty using GRADE.

**Results:**

Nine RCTs involving patients with PD were included. Pooled analysis showed no statistically significant reduction in FOG severity following external cueing interventions compared with control conditions (SMD = −0.30, 95% CI: −0.68 to 0.09), with substantial heterogeneity. In contrast, external cueing significantly improved functional mobility as measured by the TUG test (SMD = −0.65, 95% CI: −1.15 to −0.15), although heterogeneity remained high. No consistent benefits were observed for overall motor symptoms or balance outcomes. Exploratory analysis incorporating short-term follow-up data suggested a modest improvement in FOG severity; however, these findings should be interpreted with caution due to persistent heterogeneity. The certainty of evidence for all outcomes was rated as low according to the GRADE framework. The observed heterogeneity was likely attributable to variations in intervention protocols, cueing modalities, and outcome assessment methods.

**Conclusion:**

External cueing interventions may improve functional mobility in individuals with PD, particularly for tasks involving gait initiation and turning. However, current evidence does not support consistent benefits for FOG severity, overall motor symptoms, or balance. Given the low certainty of evidence, further high-quality, multicenter RCTs with standardized outcome measures and longer follow-up are needed to clarify the clinical role of cueing-based interventions in FOG. These findings highlight important limitations in the current evidence base and provide directions for future research.

**Systematic review registration:**

https://www.crd.york.ac.uk/PROSPERO/view/CRD420261280910, identifier CRD420261280910.

## 1 Introduction

Parkinson’s disease (PD) is a progressive neurodegenerative disorder that predominantly affects older adults and is clinically characterized by bradykinesia, rigidity, resting tremor, and postural instability (Bloem et al., 2021). As the disease advances, gait disturbances become increasingly prominent and represent a major source of disability. Among these disturbances, freezing of gait (FOG) is one of the most debilitating and challenging symptoms to manage. FOG is defined as a sudden, episodic inability to initiate or continue walking, typically occurring during gait initiation, turning, or when navigating narrow spaces (Spildooren et al., 2010). FOG is reported by approximately one in four individuals with PD early in the disease course, rising to a prevalence of up to 90% in the advanced stages (Cilia et al., 2015). Importantly, FOG is strongly associated with falls, reduced mobility, loss of independence, and increased mortality, imposing a substantial burden on patients, caregivers, and healthcare systems (Okuma, 2014).

Despite advances in pharmacological and surgical treatments, FOG often responds poorly to dopaminergic therapy and frequently persists during the medication “ON” state (Nonnekes et al., 2015). This limited responsiveness suggests that FOG arises from complex dysfunctions extending beyond dopamine deficiency, involving impaired integration of cognitive, sensory, and motor processes within distributed cortical–basal ganglia–cerebellar networks (Lewis et al., 2022). Aging-related declines in attentional capacity, sensorimotor integration, and neural plasticity may further exacerbate these deficits, rendering FOG particularly prevalent and refractory in older individuals with PD. Non-pharmacological treatments, such as teaching patients to use sensory cues, have been recognized as key interventions for mitigating FOG (Schaafsma et al., 2003; Lucas McKay et al., 2019).

External cueing has emerged as a key rehabilitation strategy for improving gait and mitigating FOG in PD. It is typically classified into three categories: visual cues (e.g., stripes or laser flashes projected on the floor); auditory cues (e.g., metronome beats, rhythmic music, and verbal instructions); and somatosensory cues (e.g., vibrotactile or tactile feedback). Currently, the dominant neurophysiological explanation for the efficacy of external cues is that they promote a redirection of neural activity from more impaired circuits toward relatively spared ones, effectively facilitating a shift from habitual to goal-directed behavior (Redgrave et al., 2010). By providing structured sensory information, external cues are thought to bypass impaired automatic motor control and facilitate goal-directed movement through enhanced attentional engagement and alternative neural pathways (Nieuwboer et al., 2009).

Although numerous randomized controlled trials (RCTs) have investigated the effects of external cueing on FOG and gait performance, their findings remain inconsistent. Some studies have reported reductions in FOG severity or improvements in gait parameters following auditory or visual cueing, whereas others have observed minimal or no benefit (Arias and Cudeiro, 2010; Ledger et al., 2008; Martin et al., 2015). Moreover, much of the existing literature has focused on immediate or single-session effects under laboratory conditions, limiting conclusions regarding the sustainability and real-world relevance of cueing interventions (Ginis et al., 2018; Mancini et al., 2019; Sweeney et al., 2019). Methodological heterogeneity, including differences in cueing modality, training duration, outcome measures, and participant characteristics, has further complicated evidence synthesis and contributed to conflicting results (Hulzinga et al., 2020).

Several reviews have examined cueing interventions for FOG in PD; however, most are narrative in nature or focus on a single modality, and earlier meta-analyses are now outdated or limited by language restrictions and incomplete coverage of recent high-quality RCTs (Ginis et al., 2018; Spaulding et al., 2013). To date, no comprehensive meta-analysis has systematically compared the effects of different cueing modalities using rigorous inclusion criteria, standardized risk-of-bias assessment, and formal evaluation of evidence certainty. Addressing this gap is particularly important for aging neuroscience, as identifying modality-specific benefits may inform personalized rehabilitation strategies tailored to the neural and functional profiles of older adults with PD.

Compared with previous reviews, this study emphasizes repeated cueing interventions beyond single-session effects, includes multilingual evidence, and applies updated methodological frameworks to provide a more clinically relevant and methodologically robust synthesis. Therefore, the present study conducted a systematic review and meta-analysis of randomized controlled trials to (1) evaluate the overall efficacy of repeated external cueing interventions (≥2 weeks) in reducing FOG and improving gait-related outcomes in PD, and (2) compare the effects of different cueing modalities. By synthesizing recent multilingual evidence and grading the certainty of findings, this review aims to provide a robust and clinically meaningful evidence base to guide gait rehabilitation and fall prevention in aging populations with PD.

## 2 Methods

This systematic review and meta-analysis were conducted in accordance with the Preferred Reporting Items for Systematic Reviews and Meta-Analyses (PRISMA) 2020 guidelines (Page et al., 2021). The study protocol was prospectively registered in PROSPERO (registration number: CRD420261280910).

### 2.1 Literature search strategy

A comprehensive and systematic literature search was conducted across seven electronic databases, including Web of Science, PubMed, Embase, the Cochrane Library, China National Knowledge Infrastructure (CNKI), Wanfang Database, and the VIP Database for Chinese Technical Periodicals, from their inception to November 10, 2025. No restrictions on language or publication date were applied. The search strategy combined controlled vocabulary (e.g., MeSH terms) and free-text keywords related to Parkinson’s disease, external cueing, and randomized controlled trials, using the core terms such as “Parkinson” OR “Parkinson’s disease” AND “external cue*” OR “cueing” OR “cues” AND “randomized controlled trial” OR “RCT” OR “random*.” Detailed search strategies for each database are provided in Supplementary Table 1. In addition, reference lists of included studies and relevant systematic reviews were manually screened to identify additional eligible studies.

All retrieved records were imported into EndNote (version 20), and duplicate publications were identified and removed following the deduplication method described by Bramer et al. (2016). Two independent reviewers (SQL and YWF) screened titles and abstracts according to predefined inclusion and exclusion criteria, followed by full-text assessment of potentially eligible studies. Discrepancies were resolved through discussion, and if necessary, consultation with a third reviewer (NHF).

### 2.2 Inclusion and exclusion criteria

Studies were included if they were randomized controlled trials (RCTs) enrolling adult participants with clinically confirmed Parkinson’s disease (PD) according to the UK PD Society Brain Bank diagnostic criteria (Hughes et al., 1992), without comorbid neurological disorders, and presenting with self-reported or clinically validated freezing of gait (FOG), to ensure that the study population was relevant to the target symptom of interest. Eligible interventions were repeated external cueing training (≥2 weeks) (visual, auditory, or tactile) compared with usual care, no cueing, or other non-cueing rehabilitation programs. This threshold was used to distinguish repeated training interventions from single-session or immediate cueing effects. Studies were required to report at least one quantifiable outcome related to FOG severity (e.g., FOG-Q, NFOG-Q, frequency or duration of FOG episodes) or gait and balance performance (e.g., gait speed, stride length, TUG, BBS, 6MWT, MDS-UPDRS III) with sufficient data for effect size calculation.

Studies were excluded if they were non-RCTs (e.g., quasi-experimental, single-arm, case reports, reviews, conference abstracts, protocols, qualitative studies, or animal experiments), assessed only immediate or single-session effects, used interventions not primarily based on cueing, involved non-PD or mixed neurological populations without separate PD data, or lacked extractable or complete datasets.

### 2.3 Data extraction

Data extraction was independently performed by two reviewers (SQL and YWF) using a standardized Excel-based data extraction form developed a priori. Extracted information included the first author and year of publication, country, sample size, participant characteristics (age, and Parkinson’s disease severity based on the Hoehn and Yahr stage), intervention characteristics (type of external cueing, including visual, auditory, tactile, or multimodal cueing; training duration), comparator conditions (e.g., usual care, no intervention, or wait-list control), and reported outcomes. Primary outcomes included measures of freezing of gait (FOG), such as FOG Questionnaire (FOGQ or NFOGQ) scores, number or duration of FOG episodes, and percentage of time spent in FOG. Secondary outcomes included gait parameters (e.g., step length, stride length, cadence, and walking speed) and balance or mobility measures (e.g., Timed Up and Go test). Disagreements in data extraction were resolved through discussion, and if consensus could not be reached, a third reviewer (NHF) was consulted to ensure data accuracy and consistency.

### 2.4 Quality assessment

The methodological quality and risk of bias of the included studies were independently assessed by two reviewers (SQL and NHF) using the Cochrane Risk of Bias 2.0 tool (RoB 2) (Sterne et al., 2019) for randomized trials. The RoB 2 tool evaluates five specific domains: (1) bias arising from the randomization process; (2) bias due to deviations from intended interventions; (3) bias due to missing outcome data; (4) bias in measurement of the outcome; and (5) bias in selection of the reported result. An overall risk-of-bias judgment (low, some concerns, or high) was derived for each study based on these domains. Any discrepancies between the two reviewers were resolved through discussion or, if necessary, by consultation with a third senior reviewer (YWF).

### 2.5 Grade of evidence

The overall certainty of the evidence for each critical or important outcome was evaluated using the Grading of Recommendations Assessment, Development, and Evaluation (GRADE) methodology (Guyatt et al., 2008). Two researchers (SQL and NHF) independently assessed and graded the evidence as high, moderate, low, or very low. The GRADE assessment considers five factors that may lower the certainty of evidence (risk of bias, inconsistency, indirectness, imprecision, and publication bias) and three factors that may raise it (large effect size, dose-response gradient, and plausible confounding). Disagreements in the evaluation were resolved by consensus or by consultation with a third assessor (YWF).

### 2.6 Statistical analysis

Meta-analyses were conducted when at least three studies reported data for the same outcome measure and were considered sufficiently comparable. Pooled effect estimates were calculated using random-effects models to account for between-study heterogeneity and were expressed as standardized mean differences (SMDs) with corresponding 95% confidence intervals (CIs). All statistical analyses were performed using Stata version 15.0 and RevMan version 5.4.

For randomized controlled trials employing a crossover design, only data from the first intervention period were extracted and included in the meta-analysis to minimize potential carryover effects. When outcome data were available for different types of external cueing (e.g., visual, auditory, tactile, or multimodal cueing), subgroup meta-analyses were performed to explore cue-specific effects.

Statistical heterogeneity among studies was assessed using the I^2^ statistic and interpreted as low (I^2^ < 50%), moderate (I^2^ = 50%–74%), or high (I^2^ ≥ 75%) (Higgins and Thompson, 2002; Higgins et al., 2003). Sensitivity analyses were conducted using a leave-one-out approach to evaluate the robustness of the pooled effect estimates. Publication bias was evaluated by visual inspection of funnel plots when at least ten studies were included in a meta-analysis, supplemented by Egger’s regression test to assess small-study effects. A uniform significance threshold (α = 0.05) was applied to all statistical evaluations. Given the limited number of studies available for certain outcomes, subgroup and publication bias analyses were interpreted with caution and considered exploratory.

## 3 Results

### 3.1 Literature screening

**FIGURE 1:**
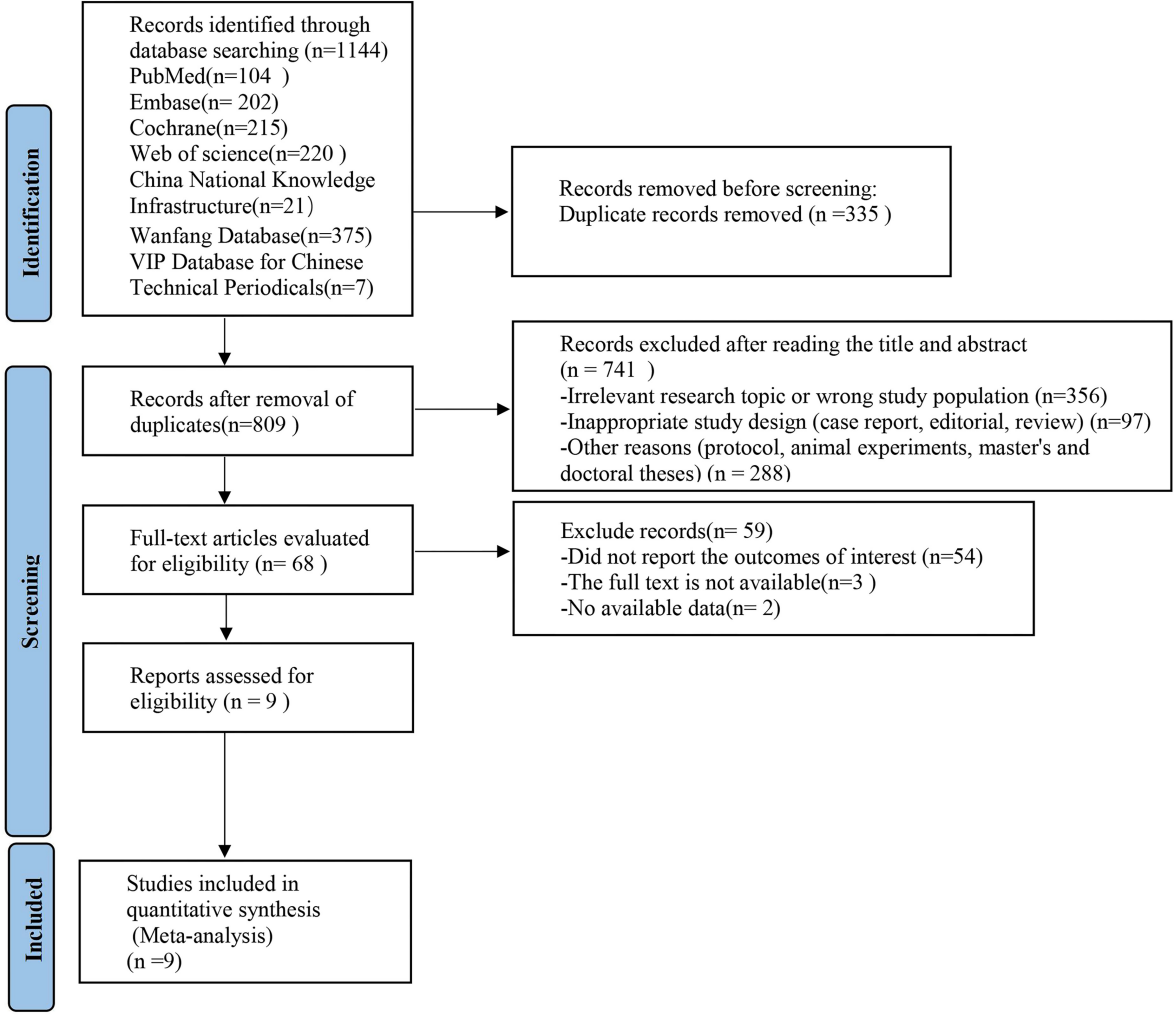
Preferred Reporting Items for Systematic review and Meta-analysis (PRISMA) flow diagram of the study process. PRISMA, Preferred Reporting Items for Systematic review and Meta-analysis.

Database searches yielded 1,144 records from sources including PubMed, Web of Science, Embase, the Cochrane Library, CNKI, Wanfang, and the VIP Database for Chinese Technical Periodicals. After removing 335 duplicates, 809 records were screened based on titles and abstracts. Based on the inclusion and exclusion criteria, 741 articles were excluded due to irrelevance, leaving 68 studies for full-text review. Following a detailed assessment, 59 studies were further excluded due to reasons such as incomplete data, non-randomized study design, or lack of appropriate outcome measures. Ultimately, nine studies met the inclusion criteria and were synthesized in the meta-analysis (see Figure 1 for PRISMA flow diagram).

### 3.2 Characteristics of included studies

A total of nine RCTs (Liu et al., 2021; Nieuwboer et al., 2007; Capato et al., 2020a, b; Zoetewei et al., 2024; Martin et al., 2015; Fietzek et al., 2014; Kim et al., 2022; Schlick et al., 2016) were included in this systematic review, investigating the effects of external cueing interventions on freezing of gait (FOG) and gait performance in patients with Parkinson’s disease (PD) (Table 1). The studies were conducted in China, Europe, South America, the Middle East, Oceania, and South Korea, with sample sizes ranging from 20 to 153 participants. The mean age of participants across studies ranged from approximately 64.2 to 78.0 years, and disease severity varied from Hoehn and Yahr stage I to IV, with most studies enrolling patients with moderate disease severity (stage II–III).

**TABLE 1:** The characteristics of included studies in the systematic review.

References	Country	Sample size (EG/CG)	Mean age, years (EG/CG)	H&Y stage	Cue modality	Intervention		Medication state	Outcomes
						EG	CG		
Liu et al. (2021)	China	127 (64/63)	72.59 ± 6.71/ 72.65 ± 6.65	NA	Auditory	Standard PD rehabilitation + intensive strength training + rhythmic auditory stimulation (1 month)	Standard PD rehabilitation (1 month)	NA	FOGQ, BBS, TUG, 6MWT
Nieuwboer et al. (2007)	UK, Belgium, Netherlands.	153 (76/77)	67.50 ± 7.78/ 69.00 ± 7.78	II–IV	Mixed	Home-based cueing training (auditory, visual, or somatosensory; 3 weeks)	Wait-list	On phase	PG score, FOGQ, gait speed, step length, TUG, FES, falls
Capato et al. (2020a)	Brazil	35 (17/18)	77.00 ± 7.00/ 78.00 ± 10.00	IV	Auditory	Multimodal balance training + rhythmic auditory stimulation (5 weeks)	Multimodal balance training (5 weeks)	On phase	BBS, TUG, NFOGQ, MDS-UPDRS III, FES-I
Capato et al. (2020b)	Brazil	1.41 (19/22) 2.40 (18/22)	1. .74 ± 8/ 76 ± 7 2. .63 ± 13/ 76 ± 7	II–III	1. Visual + auditory 2. Visual	1. RAS-supported multimodal balance training (metronome-provided auditory stimuli); 2. Regular multimodal training (without rhythmical auditory stimuli) Total intervention: 5 weeks.	Educational program (5 weeks)	On phase	TUGT, NFOGQ
Zoetewei et al. (2024)	Israel, Belgium	63 (32/31)	67.70 ± 7.97/ 68.70 ± 7.31	I–IV	Auditory	On-demand auditory cueing (DeFOG system; 4 weeks)	DeFOG system without cueing (4 weeks)	ON and OFF phase	NFOGQ, TUG, MDS-UPDRS III, %TF (total percentage time frozen), total duration of FOG, Number of FOG
Martin et al. (2015)	New Zealand	21 (12/9)	72.00 ± 5.10/ 72.00 ± 5.80	II–III	Auditory	Metronome-based auditory cueing (6 months)	Wait list	NA	NFOGQ, falls
Fietzek et al. (2014)	Germany	22 (14/8)	69.80 ± 6.52/ 64.20 ± 5.88	II–III	Mixed	Auditory, visual, or multimodal cueing (2 weeks)	Wait list	On phase	Freezing score, FOGQ
Kim et al. (2022)	South Korea	44 (22/22)	68.70 ± 6.90/ 67.50 ± 9.30	II–III	Visual + auditory	Robot-assisted gait training with auditory and visual cues (4 weeks)	Treadmill training (4 weeks)	On phase	NFOGQ, TUG, BBS, MDS-UPDRS III
Schlick et al. (2016)	Germany	20 (10/10)	71.20 ± 10.9/ 68.90 ± 6.80	II–III	Visual	Treadmill training with visual cues (6 weeks)	Treadmill training (6 weeks)	On phase	FOGQ, UPDRS III TUG, gait speed, Stride length

EG, experimental group; CG, control group; H&Y, Hoehn & Yahr; M/F, Male/Female; FOG, freezing of gait; NFOGQ, new freezing of gait questionnaire; FOGQ, freezing of gait questionnaire; DeFOG, on-demand cueing device [The DeFOG-system (mHT; Bologna, Italy) consisted of two inertial measurement units (IMUs) attached to the shoes (recording at 200 Hz) and connected to a smartphone using Bluetooth]; MDS-UPDRS, Movement Disorder Society-Unified Parkinson’s Disease Rating Scale; PDQ-39, the Parkinson’s disease questionnaire; TUG, Timed Up and Go Test; BBS, Berg balance Scale; 6MWT, 6 minute walk test; PG score, posture and gait (PG) score; FES, Falls Efficacy Scale; FES-I, Falls Efficacy Scale-International; N/A, Not Available; NR, Not reported.

The intervention duration ranged from 2 weeks to 6 months. External cueing modalities included auditory, visual, and multimodal cueing, either delivered as standalone interventions or integrated into rehabilitation programs such as balance training, treadmill training, home-based cueing, or robot-assisted gait training. Most studies assessed outcomes in the ON-medication state, while one study evaluated participants in both ON and OFF states. Commonly reported outcomes included FOG severity (FOG-Q or NFOG-Q), gait and mobility measures (Timed Up and Go test, gait speed, stride length, and 6-minute walk test), balance performance (Berg Balance Scale), and motor severity (MDS–UPDRS part III).

### 3.3 Risk of bias

**FIGURE 2:**
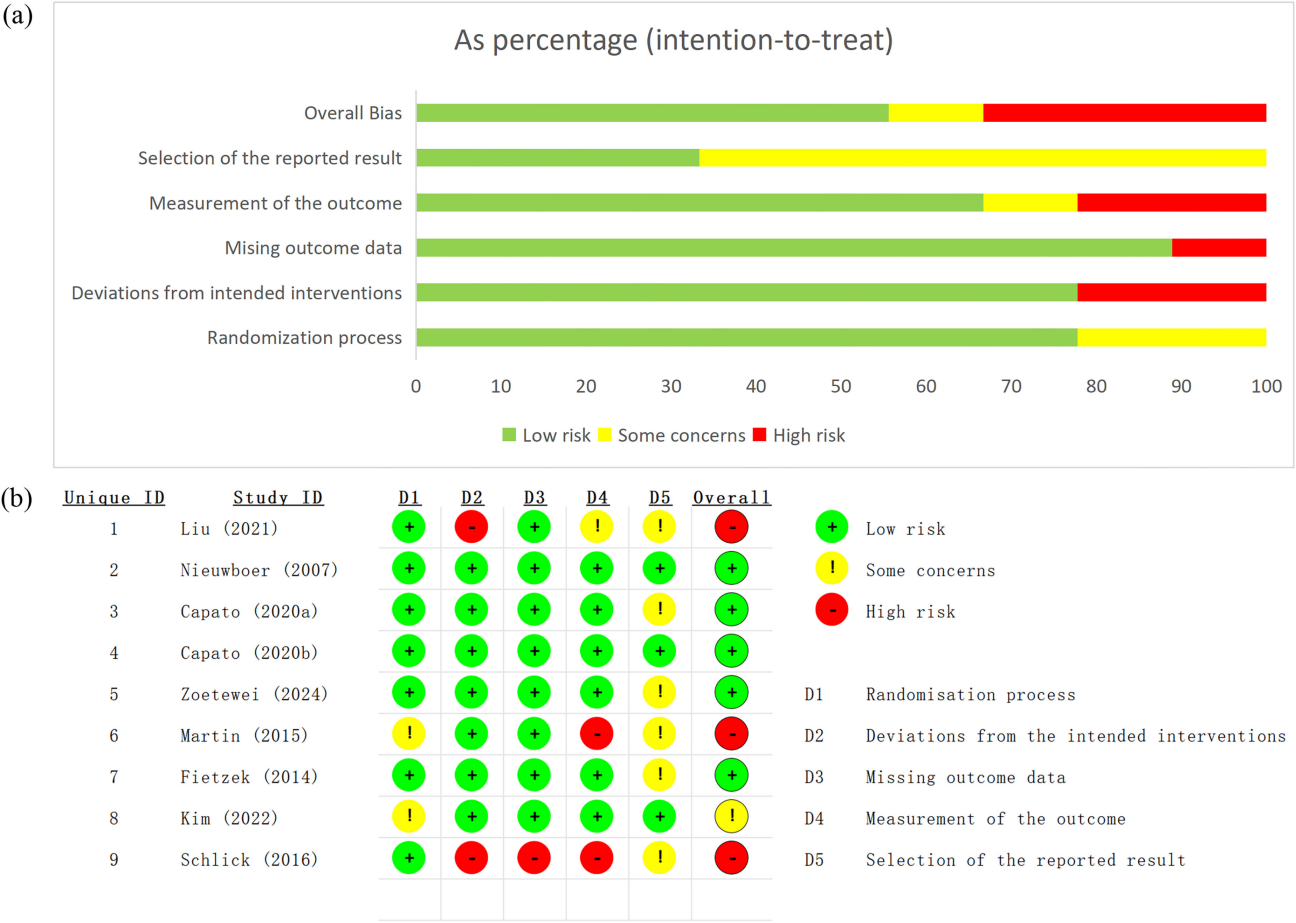
Assessment of risk of bias. **(a)** Risk of bias graph; **(b)** risk of summary.

According to the ROB 2.0 assessment, among the included studies, most were rated as having low risk of bias, while several studies were judged to have some concerns, and three studies (Liu et al., 2021; Martin et al., 2015; Schlick et al., 2016) were classified as being at high risk of bias (Figure 2).

In the randomization process, most studies were assessed as having a low risk of bias; however, some concerns were identified in several trials due to insufficient reporting of random sequence generation or allocation concealment (Martin et al., 2015; Kim et al., 2022).

Regarding deviations from intended interventions, the majority of studies were judged to be at low risk of bias. Two studies (Liu et al., 2021; Schlick et al., 2016) were rated as having a high risk of bias in this domain, primarily because deviations from the intended interventions could not be adequately ruled out.

In terms of missing outcome data, most studies showed a low risk of bias, as outcome data were largely complete or missing data were unlikely to have a meaningful impact on the results. One study (Schlick et al., 2016) was assessed as having a high risk of bias due to incomplete outcome data.

For the measurement of the outcome, most studies were considered to be at low risk of bias; however, two studies (Schlick et al., 2016; Martin et al., 2015) were judged to be at high risk, primarily due to the lack of blinded outcome assessment.

With respect to selection of the reported result, several studies were rated as having some concerns because protocol pre-registration was not reported or sufficient information was unavailable to exclude selective reporting.

### 3.4 Meta-analysis results

#### 3.4.1 Effects of external cueing on freezing of gait severity (FOGQ/NFOGQ)

Freezing of gait severity was assessed using the Freezing of Gait Questionnaire (FOGQ) or the New Freezing of Gait Questionnaire (NFOGQ). As these instruments evaluate the same clinical construct, outcomes derived from FOGQ and NFOGQ measures were pooled and analyzed together using standardized mean differences.

**FIGURE 3:**
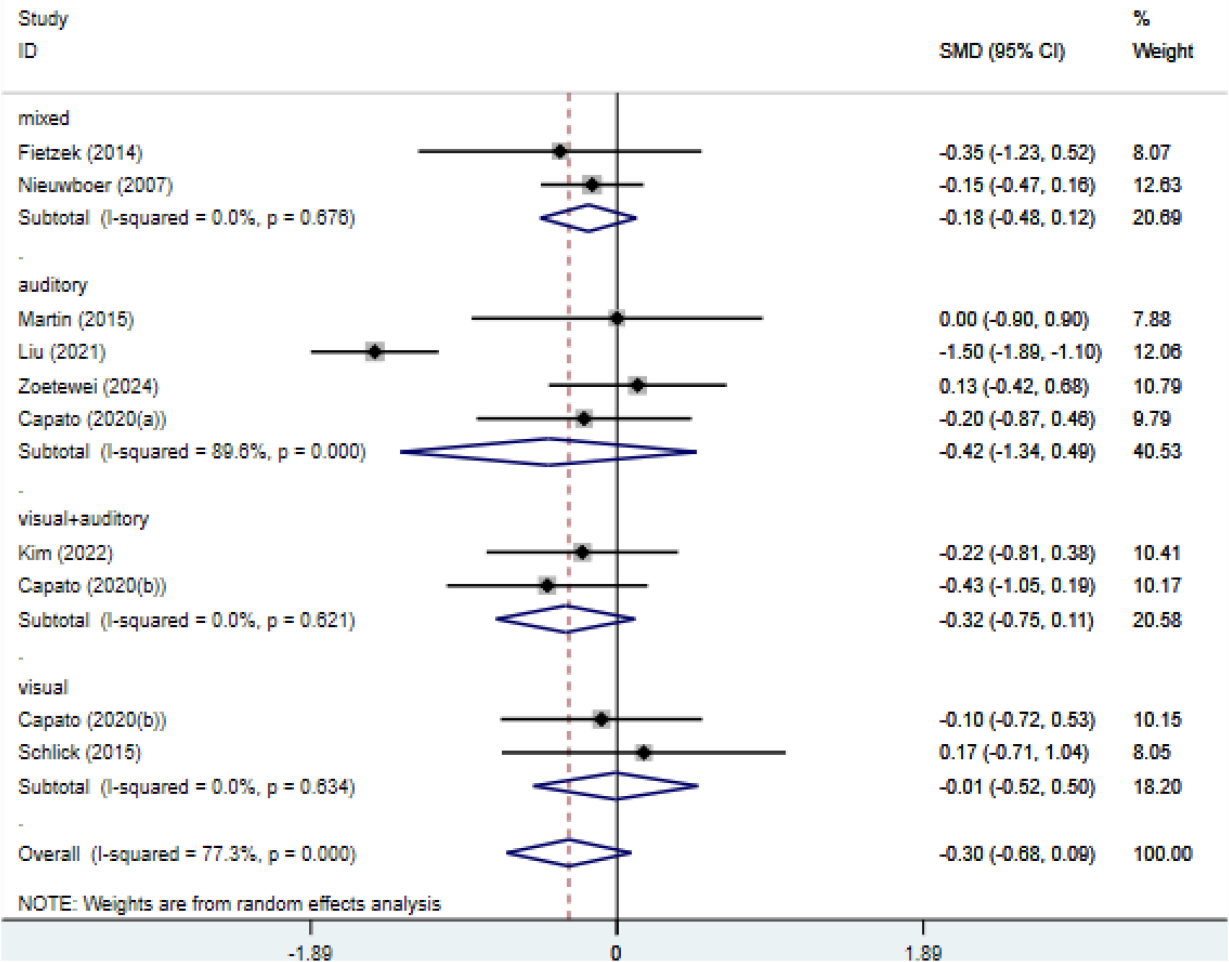
Effects of external cueing on freezing of gait severity assessed by FOGQ/NFOGQ at post-intervention. NFOGQ, new freezing of gait questionnaire; FOGQ, freezing of gait questionnaire; CI, confidence interval; SMD, standardized mean difference.

Based on post-intervention assessments, the primary meta-analysis including all eligible studies showed no statistically significant difference in freezing of gait severity between the external cueing and control groups (random-effects model; SMD = −0.30, 95% CI: −0.68 to 0.09; *P* = 0.13; I^2^ = 77.3%). Subgroup analyses according to cueing modality (auditory, visual, and multimodal cueing) did not reveal a statistically significant effect in any subgroup (Figure 3).

An exploratory analysis was conducted to examine short-term follow-up effects (approximately 1 month) where such data were available, in addition to the primary post-intervention analysis. This exploratory analysis suggested a modest reduction in freezing of gait severity favoring external cueing interventions (SMD = −0.32, 95% CI: −0.60 to −0.03; *P* = 0.03; I^2^ = 68.4%) (Supplementary Figure 1); however, this finding should be interpreted with caution given the limited number of studies, residual heterogeneity, and the exploratory nature of the analysis, as follow-up outcomes reflect short-term maintenance effects rather than immediate treatment effects.

#### 3.4.2 Meta-analysis of change in MDS-UPDRS III

**FIGURE 4:**
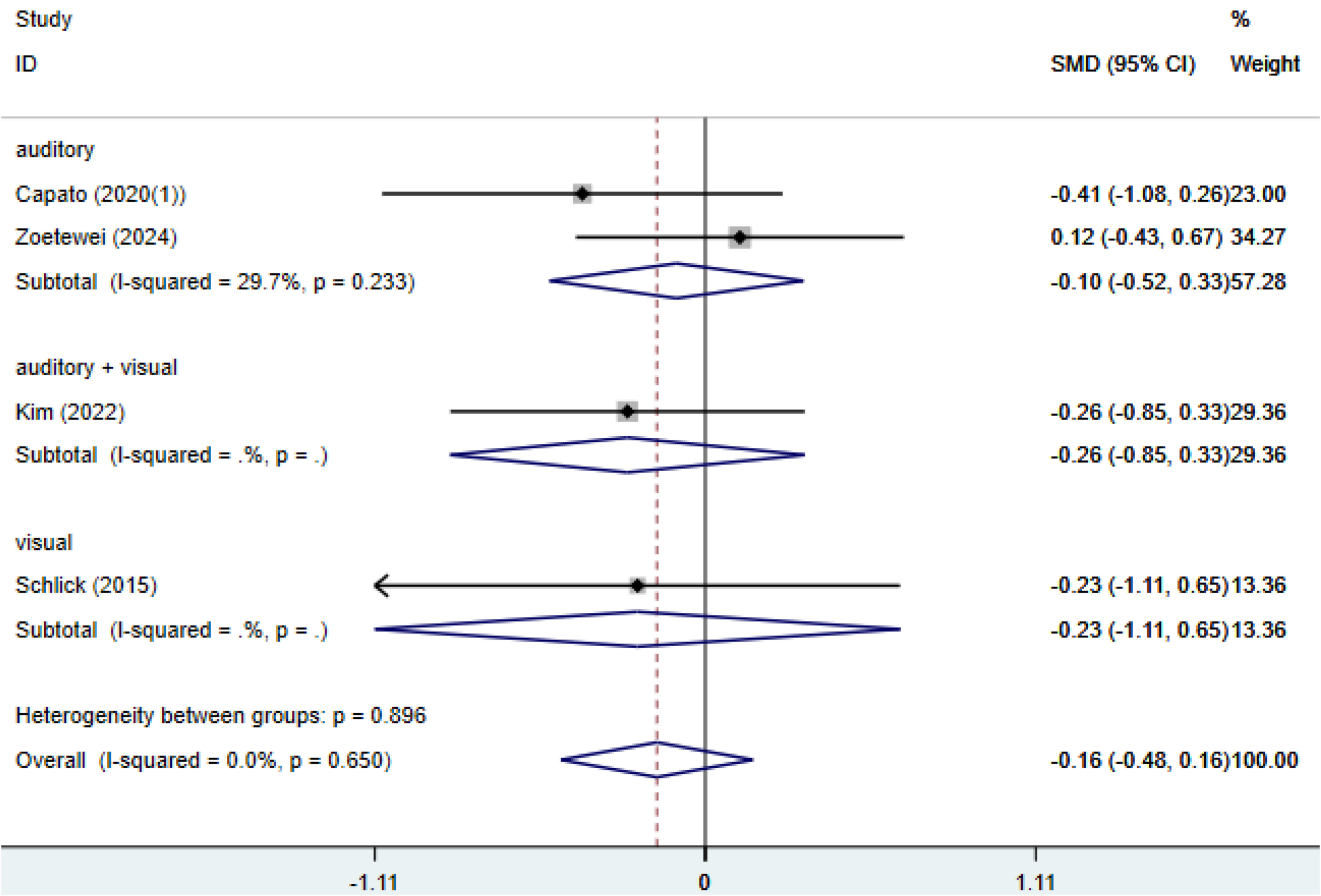
Meta-analysis of the effects of external cueing on motor symptoms assessed by MDS-UPDRS III. MDS-UPDRS, Movement Disorder Society-Unified Parkinson’s Disease Rating Scale; CI, confidence interval; SMD, standardized mean difference.

Four studies assessed the effects of cueing interventions on motor symptoms measured by the MDS-UPDRS III, including 74 participants in the cueing group and 76 in the control group. No significant heterogeneity was observed across studies (I^2^ = 0.0%, *p* = 0.90); therefore, a fixed-effects model was applied. The pooled analysis did not demonstrate a statistically significant improvement in motor function favoring cueing interventions, with a standardized mean difference (SMD) of −0.16 (95% CI: −0.48 to 0.16, *p* = 0.65) (Figure 4). Egger’s regression test indicated no evidence of publication bias (*p* = 0.50) (Supplementary Figure 2).

#### 3.4.3 Meta-analysis of change in Timed Up and Go test (TUG)

Seven randomized controlled trials evaluated the effects of external cueing interventions on motor performance assessed by the Timed Up and Go (TUG) test, including 251 participants in the experimental group and 260 participants in the control group. Substantial heterogeneity was observed among the included studies (I^2^ = 85.3%, *p* < 0.001); therefore, a random-effects model was applied.

**FIGURE 5:**
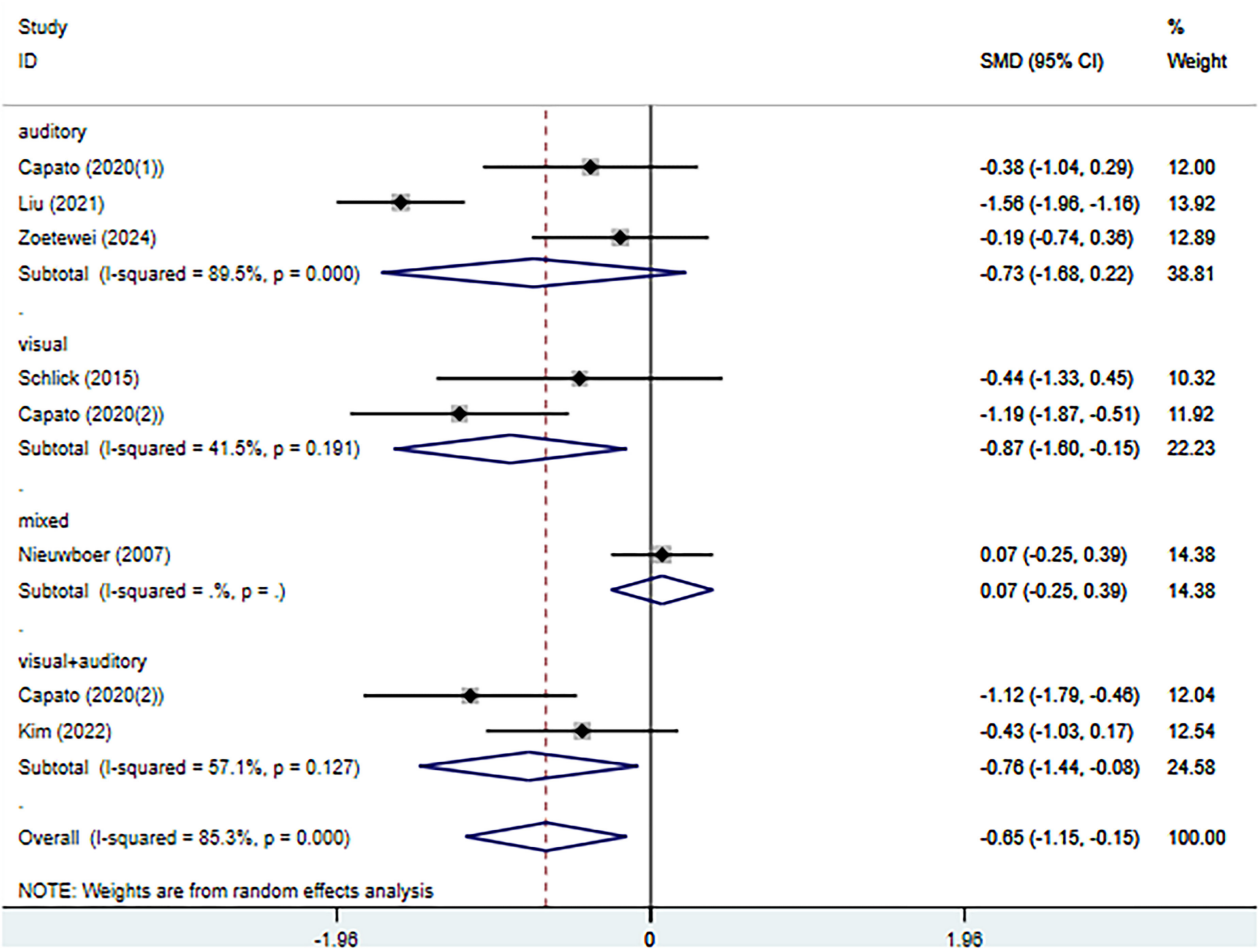
Meta-analysis of the effects of external cueing on Timed Up and Go test. CI, confidence interval; SMD, standardized mean difference.

The pooled analysis demonstrated a statistically significant improvement in TUG performance favoring external cueing interventions compared with control conditions (SMD = −0.65, 95% CI: −1.15 to −0.15; *p* = 0.01) (Figure 5). Sensitivity analysis, performed by sequentially omitting individual studies, showed that the overall effect estimate remained stable, indicating that the pooled result was robust and not driven by any single study (Supplementary Figure 3). Assessment of publication bias using Egger’s regression test revealed no significant evidence of small-study effects (*p* = 0.57) (Supplementary Figure 4).

#### 3.4.4 Meta-analysis of change in Berg Balance Scale (BBS)

Three randomized controlled trials reported balance outcomes assessed using the Berg Balance Scale (BBS), including 103 participants in the experimental group and 103 participants in the control group. Substantial heterogeneity was observed among the included studies (I^2^ = 90.2%, *p* < 0.001); therefore, a random-effects model was applied.

**FIGURE 6:**
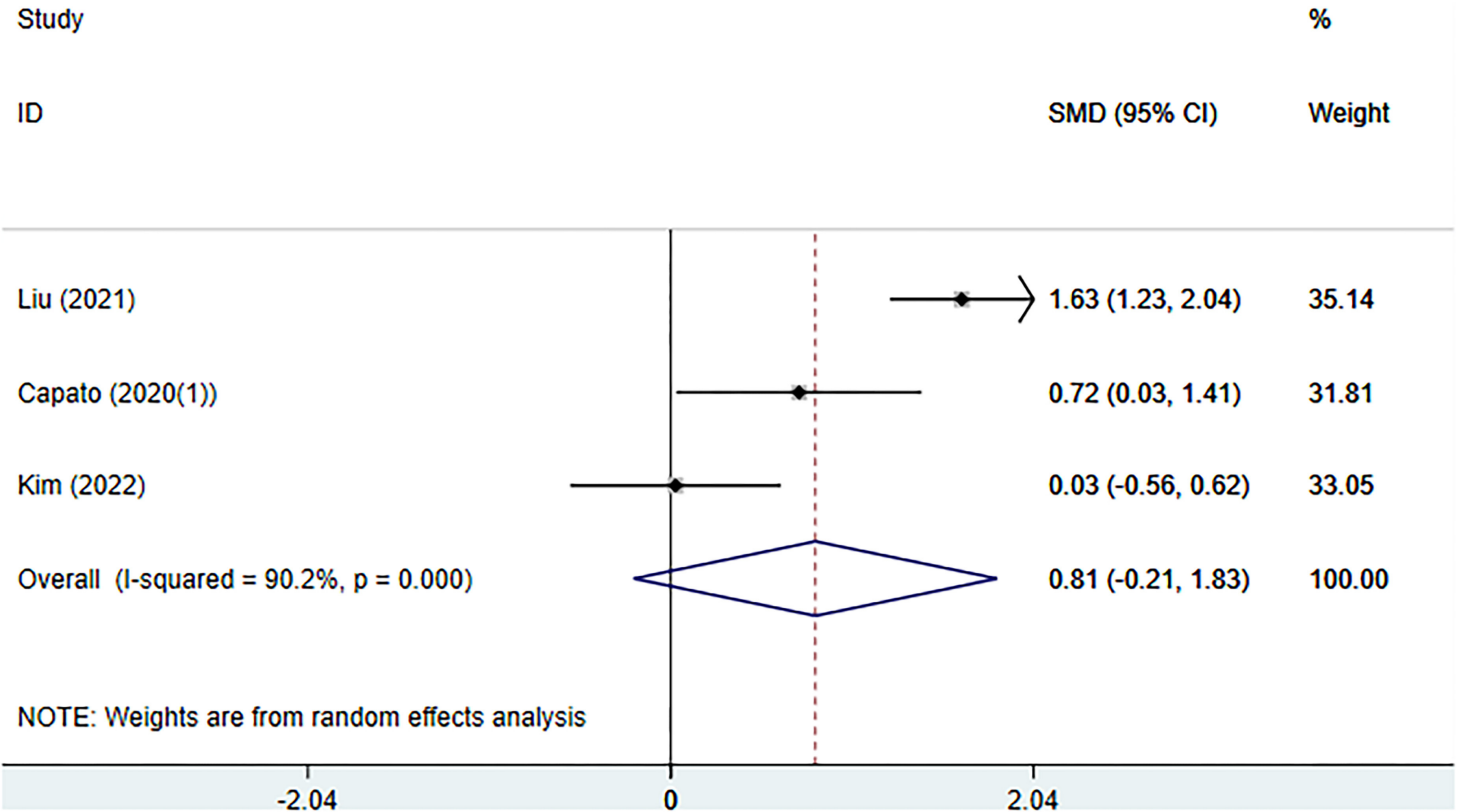
Meta-analysis of the effects of external cueing on balance performance assessed by the Berg Balance Scale.

The pooled meta-analysis showed no statistically significant difference in BBS scores between the external cueing and control groups (SMD = 0.81, 95% CI: −0.21 to 1.83; *p* = 0.12) (Figure 6). Sensitivity analysis, conducted by sequentially omitting individual studies, demonstrated that the overall effect estimate remained stable, indicating that the pooled result was robust and not driven by any single study (Supplementary Figure 5).

Given that fewer than ten studies were included, formal assessment of publication bias was not performed for the BBS outcome.

### 3.5 Sensitivity analysis for FOGQ/NFOGQ outcomes

Sensitivity analyses were performed to assess the robustness of the pooled effect estimates for FOGQ/NFOGQ outcomes. Sequential omission of individual studies did not materially alter the direction or magnitude of the pooled effect size. In addition, analyses excluding follow-up data and retaining only post-intervention measurements yielded consistent effect estimates, indicating that the overall findings were not driven by a single study or a specific assessment time point (Supplementary Figure 6).

### 3.6 Publication bias for FOGQ/NFOGQ outcomes

Publication bias for the FOGQ/NFOGQ outcomes was assessed using Egger’s regression test. The test did not indicate significant small-study effects (*p* = 0.37) (Supplementary Figure 7). Given the limited number of included studies, the results of the publication bias assessment should be interpreted with caution.

### 3.7 GRADE evidence profile for the studies in the meta-analysis

Based on the GRADE approach, the certainty of evidence across all outcomes was assessed as low (Table 2).

**TABLE 2:** Grading of Recommendations Assessment, Development, and Evaluation (GRADE) evidence profile for the studies in the meta-analysis.

Certainty assessment							No. of patients		Effect		Certainty
No. of studies	Study design	Risk of bias	Inconsistency	Indirectness	Imprecision	Other considerations	External cueing training	Non-cueing rehabilitation programs	Relative (95% CI)	Absolute (95% CI)	
FOG severity (FOGQ/NFOGQ)											
9	Randomized trials	Serious^a^	Not serious	Not serious	Serious^b^	None	275	277	–	SMD 0.3 SD lower (0.68 lower to 0.09 higher)	⊕⊕oo Low^a,b^
MDS-UPDRS III											
4	Randomized trials	Serious^a^	Not serious	Not serious	Serious^b^	None	74	76	–	SMD 0.16 SD lower (0.48 lower to 0.16 higher)	⊕⊕oo Low^a,b^
TUG											
7	Randomized trials	Serious^a^	Not serious	Not serious	Serious^b^	None	251	260	–	SMD 0.65 SD lower (1.15 lower to 0.15 lower)	⊕⊕oo Low^a,b^
BBS											
3	Randomized trials	Serious^a^	Not serious	Not serious	Serious^b^	None	103	103	–	SMD 0.81 SD higher (0.21 lower to 1.83 higher)	⊕⊕oo Low^a,b^

CI, confidence interval; SMD, standardized mean difference; FOG, freezing of gait; NFOGQ, new freezing of gait questionnaire; FOGQ, freezing of gait questionnaire; MDS-UPDRS, Movement Disorder Society-Unified Parkinson’s Disease Rating Scale; TUG, Timed Up and Go Test; BBS, Berg balance Scale. ^a^Downgraded one level due to risk of bias: most included RCTs lacked blinding of participants and outcome assessors, and several did not clearly report methods of allocation concealment. ^b^Downgraded one level due to imprecision: the total sample size across included studies was small. ⊕ Represents a unit of high-quality evidence, and o represents a unit of downgraded evidence, together indicating the GRADE certainty level (⊕⊕⊕⊕ high, ⊕⊕⊕o moderate, ⊕⊕oo low, ⊕ooo very low).

## 4 Discussion

This systematic review and meta-analysis synthesized evidence from nine randomized controlled trials to evaluate the effects of external cueing interventions on freezing of gait (FOG) and gait-related outcomes in individuals with Parkinson’s disease. Overall, the findings indicate that external cueing provides task-specific benefits for functional mobility, particularly as reflected by improvements in Timed Up and Go (TUG) performance, whereas consistent effects on FOG severity, global motor symptoms, and balance were not demonstrated. These findings should be interpreted in the context of a limited number of randomized controlled trials and substantial heterogeneity across studies.

The pooled analysis of FOGQ and NFOGQ scores did not show a statistically significant reduction in FOG severity immediately after intervention, and substantial heterogeneity was observed across studies. This finding aligns with previous systematic reviews reporting inconsistent effects of cueing on FOG severity and highlights the challenges of using self-reported questionnaires to capture episodic and context-dependent freezing phenomena (Kwok et al., 2022; Cosentino et al., 2023). In particular, Hulzinga et al. (2020) questioned the responsiveness of the NFOG-Q as a primary outcome in clinical trials, noting potential self-report bias and limited sensitivity to change. Therefore, the lack of significant findings may partly reflect limitations of subjective outcome measures rather than the absence of a true intervention effect, highlighting the need for objective assessments in future studies.

Although an exploratory analysis incorporating short-term follow-up data suggested a modest benefit of cueing on FOG severity, this result should be interpreted with caution given the limited number of studies, residual heterogeneity, and the fact that follow-up outcomes reflect short-term maintenance effects rather than immediate treatment effects. Therefore, direct comparisons between post-intervention and follow-up findings should be made cautiously.

Subgroup analyses based on cueing modality (auditory, visual, and multimodal) did not reveal a statistically significant advantage for any specific approach. However, this lack of statistically significant differences between modalities should not be interpreted as evidence of equivalence, but may reflect limited statistical power and variability in intervention protocols. This is consistent with prior evidence suggesting that cueing primarily acts as a compensatory strategy by engaging attentional control mechanisms rather than directly restoring impaired automatic gait control (Ginis et al., 2018; Nieuwboer et al., 2009; Kwok et al., 2022). The substantial heterogeneity observed across outcomes is likely multifactorial. In addition to differences in cueing modality, variability in intervention duration (ranging from 2 weeks to 6 months), types of control conditions (e.g., usual care vs. active rehabilitation), timing of outcome assessment (immediate post-intervention vs. follow-up), and participant characteristics (e.g., disease severity and presence of freezing of gait) may have contributed to the observed inconsistency. Furthermore, methodological differences, including variations in outcome measurement tools and study design, may have also influenced the results. Such heterogeneity is not unexpected in rehabilitation research, where interventions are inherently complex and context-dependent. This heterogeneity reflects variability in current study designs and highlights the need for more standardized protocols in future research. Given the limited number of studies available for each outcome, further subgroup analyses or meta-regression were not feasible without risking unreliable estimates. Therefore, the current findings regarding subgroup effects should be interpreted with caution, and these potential sources of heterogeneity should be further explored in future well-designed studies.

In contrast to FOG severity outcomes, external cueing was associated with a significant improvement in TUG performance, indicating enhanced functional mobility. This apparent discrepancy between objective and subjective outcomes may be explained by several factors. First, the Timed Up and Go (TUG) test is an objective, performance-based measure that captures specific components of mobility, including gait initiation, turning, and transitional movements, situations in which freezing of gait is most likely to occur. External cueing may facilitate these task-specific motor processes by providing structured sensory input and enhancing attentional control, thereby improving performance during standardized testing conditions. In contrast, the Freezing of Gait Questionnaire (FOGQ/NFOGQ) is a subjective, self-reported measure that reflects patients’ perceptions of freezing episodes in daily life. Such measures may be less sensitive to short-term changes and are potentially influenced by recall bias, symptom variability, and patients’ awareness of freezing events. As a result, improvements observed in controlled clinical tasks may not be fully captured by questionnaire-based assessments. Moreover, external cueing is generally considered a compensatory strategy that facilitates goal-directed movement rather than directly modifying the underlying pathophysiology of freezing of gait. Therefore, while cueing may enhance functional performance in structured tasks such as the TUG, it may not necessarily translate into a consistent reduction in the frequency or severity of freezing episodes in real-world settings. This distinction highlights the importance of selecting appropriate outcome measures and suggests that objective and subjective assessments may capture different dimensions of treatment response. This interpretation is consistent with previous studies reporting task-specific benefits of cueing in complex mobility tasks rather than generalized motor improvement (Spaulding et al., 2013; Nieuwboer et al., 2007).

No statistically significant effects were observed for MDS-UPDRS part III or Berg Balance Scale outcomes. These findings suggest that the benefits of cueing may not generalize to overall motor severity or static balance function. Moreover, the limited number of studies and substantial heterogeneity for balance outcomes further restrict the interpretability of these results.

From a methodological perspective, although most included studies were judged to be at low risk of bias in several domains, concerns remained regarding blinding, selective reporting, and small sample sizes. Accordingly, the certainty of evidence for all outcomes was rated as low according to the GRADE approach, mainly due to heterogeneity and imprecision. These limitations underscore the need for cautious interpretation of the findings. Another important limitation of this meta-analysis is the relatively small number of included randomized controlled trials, particularly for specific outcome measures. This may reduce statistical power and increase uncertainty in the pooled estimates. Although broader inclusion criteria could have increased the number of studies, we deliberately restricted our analysis to repeated cueing interventions to enhance methodological rigor and clinical relevance. Furthermore, subgroup analyses and publication bias assessments were conducted in the context of a limited number of studies, and their results should therefore be interpreted as exploratory rather than confirmatory. Future large-scale, high-quality randomized trials are needed to provide more robust evidence.

Importantly, this study contributes to the current literature in several ways. First, it provides a synthesis of randomized evidence on repeated cueing interventions. Second, it highlights the discrepancy between improvements in functional mobility and inconsistent effects on FOG severity. Third, it identifies key methodological limitations, including small sample sizes, heterogeneity, and reliance on subjective outcome measures. Finally, it outlines priorities for future research, including modality-specific trials and the use of objective outcome measures. Recent advances in personalized and on-demand cueing systems may offer new directions for intervention development. One possible explanation lies in the mode and timing of cue delivery. Traditional cueing interventions in the included studies were typically administered continuously during training sessions, which may reduce attentional engagement over time due to habituation. As cues become predictable, their ability to effectively trigger motor responses may diminish, particularly in individuals with impaired attentional control. In contrast, on-demand cueing systems deliver stimuli precisely when freezing episodes are detected or anticipated. This event-driven approach may enhance cue salience and facilitate more effective motor compensation during critical moments. Given that freezing of gait is episodic and context-dependent, interventions aligned with real-time motor fluctuations may be more effective than continuous cueing strategies. These considerations may help explain why repeated cueing training demonstrated limited effects on FOG severity in this meta-analysis, whereas emerging studies on adaptive cueing have reported more promising results. For example, Zoetewei et al. (2024) evaluated an on-demand auditory cueing system (DeFOG) and reported moderate within-group improvements, although between-group differences were not statistically significant. While preliminary, these findings suggest that adaptive, context-sensitive cueing approaches may overcome some limitations of conventional continuous cueing. Future research should further investigate personalized strategies that optimize timing, modality, and individual responsiveness.

## 5 Conclusion

Rather than providing definitive conclusions, this study highlights important gaps in the current evidence base and underscores the need for high-quality, standardized randomized controlled trials. This meta-analysis indicates that external cueing interventions may improve functional mobility, as reflected by significant benefits in Timed Up and Go performance, in individuals with Parkinson’s disease. However, current evidence does not demonstrate consistent improvements in freezing of gait severity, overall motor symptoms, or balance outcomes. The certainty of evidence across outcomes remains low, largely due to heterogeneity, small sample sizes, and methodological limitations. Further high-quality, multicenter randomized controlled trials using standardized and objective measures, longer follow-up periods, and adaptive cueing strategies are required to clarify the clinical role of external cueing in the management of freezing of gait.

## Data Availability

The raw data supporting the conclusions of this article will be made available by the authors, without undue reservation.
