# Supplementary material for: Efficacy of repeated external cueing training on freezing of gait and gait performance in Parkinson’s disease: a systematic review and meta-analysis
Source: Front Aging Neurosci. 2026 May 13;18:1808824. doi: 10.3389/fnagi.2026.1808824 (PMC13212247; doi:10.3389/fnagi.2026.1808824)
Supplement: Supplementary file 1 [file Data_Sheet_1.docx]

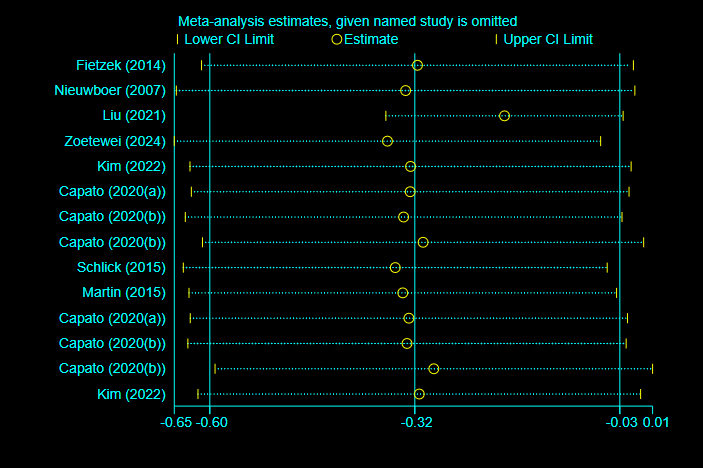


**Figure S1. Sensitivity analysis of external cueing effects on freezing of gait severity incorporating follow-up data. CI: confidence interval.**


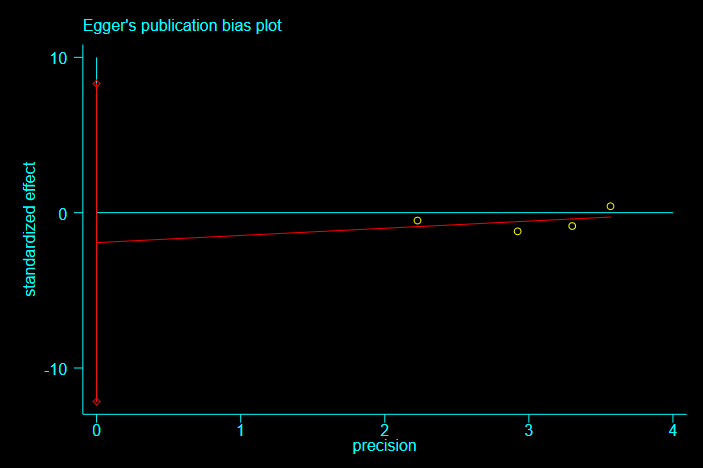


**Figure S2. Assessment of publication bias for MDS-UPDRS III outcomes.**

**MDS-UPDRS: Movement Disorder Society-Unified Parkinson’s Disease Rating Scale.**


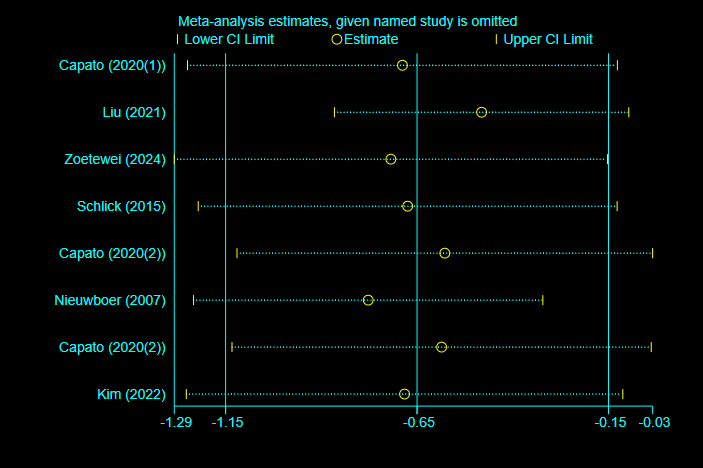


**Figure S3. Sensitivity analysis of the TUG outcome. TUG:Timed Up and Go Test; CI: confidence interval.**


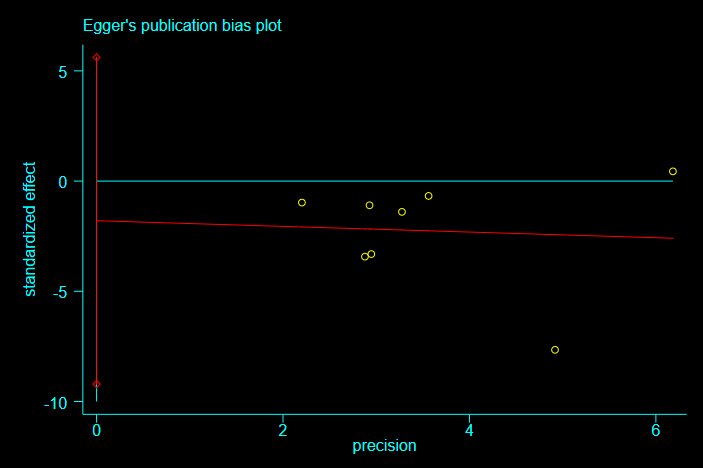


**Figure S4. Assessment of publication bias for the TUG outcome. TUG:Timed Up and Go Test.**


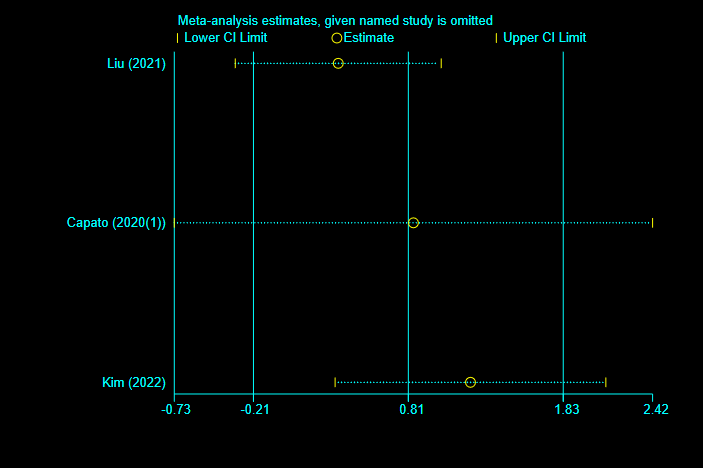


**Figure S5. Sensitivity analysis of the BBS outcome. BBS: Berg balance Scale; CI: confidence interval.**


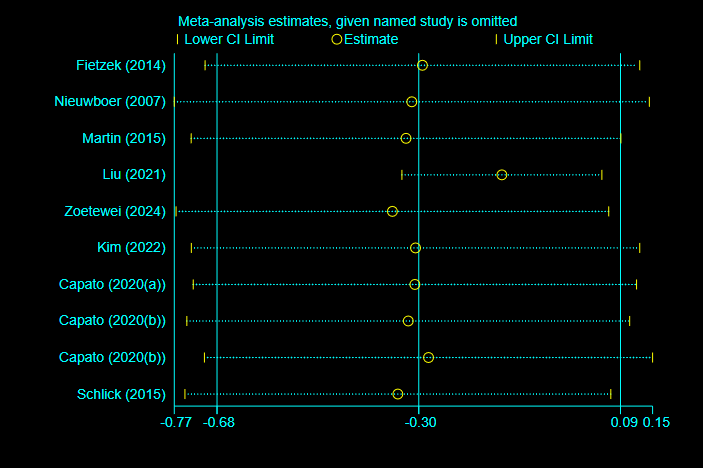


**Figure S6. Sensitivity analysis for FOGQ/NFOGQ outcomes.**

**FOGQ: freezing of gait questionnaire; NFOGQ: new freezing of gait questionnaire; CI: confidence interval.**


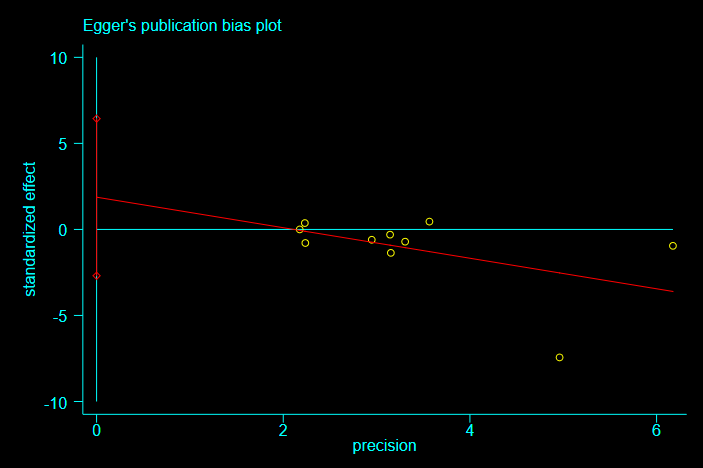


**Figure S7. Assessment of publication bias for FOGQ/NFOGQ outcomes.**

**FOGQ: freezing of gait questionnaire; NFOGQ: new freezing of gait questionnaire.**

**Table S1 Full Electronic Search Strategies in Each Database**

| **Database** | **Search number** | **Search Strategy** | **Date of Search** | **Records Retrieved** |
| --- | --- | --- | --- | --- |
| PubMed | 10 | ("Parkinson Disease"[MeSH Terms] OR ("Parkinson Disease"[Title/Abstract] OR "idiopathic parkinson disease"[Title/Abstract] OR "idiopathic parkinson s disease"[Title/Abstract] OR "lewy body parkinson disease"[Title/Abstract] OR "lewy body parkinson s disease"[Title/Abstract] OR "paralysis agitans"[Title/Abstract] OR "parkinson disease idiopathic"[Title/Abstract] OR "parkinson s disease"[Title/Abstract] OR "parkinson s disease idiopathic"[Title/Abstract] OR "parkinson s disease lewy body"[Title/Abstract] OR "primary parkinsonism"[Title/Abstract] OR "parkinsonism primary"[Title/Abstract])) AND ("Cues"[MeSH Terms] OR ("Cues"[Title/Abstract] OR "Cueing"[Title/Abstract] OR "Cue"[Title/Abstract] OR "rhythmic auditory stimulation"[Title/Abstract] OR "Vibration"[Title/Abstract] OR "external rhythmic stimulation"[Title/Abstract])) AND ("Randomized Controlled Trials as Topic"[MeSH Terms] OR ("clinical trials randomized"[Title/Abstract] OR "trials randomized clinical"[Title/Abstract] OR "controlled clinical trials randomized"[Title/Abstract] OR "randomised controlled study"[Title/Abstract] OR "randomised controlled trial"[Title/Abstract] OR "randomized controlled study"[Title/Abstract] OR "randomized controlled trial"[Title/Abstract] OR "Random"[Title/Abstract] OR "RCT"[Title/Abstract] OR "randomized experiment"[Title/Abstract])) | 10-Nov-2025 | 104 |
|  | 9 | "Randomized Controlled Trials as Topic"[MeSH Terms] OR "clinical trials randomized"[Title/Abstract] OR "trials randomized clinical"[Title/Abstract] OR "controlled clinical trials randomized"[Title/Abstract] OR "randomised controlled study"[Title/Abstract] OR "randomised controlled trial"[Title/Abstract] OR "randomized controlled study"[Title/Abstract] OR "randomized controlled trial"[Title/Abstract] OR "Random"[Title/Abstract] OR "RCT"[Title/Abstract] OR "randomized experiment"[Title/Abstract] | 10-Nov-2025 | 789,393 |
|  | 8 | "clinical trials randomized"[Title/Abstract] OR "trials randomized clinical"[Title/Abstract] OR "controlled clinical trials randomized"[Title/Abstract] OR "randomised controlled study"[Title/Abstract] OR "randomised controlled trial"[Title/Abstract] OR "randomized controlled study"[Title/Abstract] OR "randomized controlled trial"[Title/Abstract] OR "Random"[Title/Abstract] OR "RCT"[Title/Abstract] OR "randomized experiment"[Title/Abstract] | 10-Nov-2025 | 641,527 |
|  | 7 | "Randomized Controlled Trials as Topic"[MeSH Terms] | 10-Nov-2025 | 192,433 |
|  | 6 | "Cues"[MeSH Terms] OR "Cues"[Title/Abstract] OR "Cueing"[Title/Abstract] OR "Cue"[Title/Abstract] OR "rhythmic auditory stimulation"[Title/Abstract] OR "Vibration"[Title/Abstract] OR "external rhythmic stimulation"[Title/Abstract] | 10-Nov-2025 | 180,046 |
|  | 5 | "Cues"[Title/Abstract] OR "Cueing"[Title/Abstract] OR "Cue"[Title/Abstract] OR "rhythmic auditory stimulation"[Title/Abstract] OR "Vibration"[Title/Abstract] OR "external rhythmic stimulation"[Title/Abstract] | 10-Nov-2025 | 165,672 |
|  | 4 | "Cues"[MeSH Terms] | 10-Nov-2025 | 45,616 |
|  | 3 | "Parkinson Disease"[MeSH Terms] OR "Parkinson Disease"[Title/Abstract] OR "idiopathic parkinson disease"[Title/Abstract] OR "idiopathic parkinson s disease"[Title/Abstract] OR "lewy body parkinson disease"[Title/Abstract] OR "lewy body parkinson s disease"[Title/Abstract] OR "paralysis agitans"[Title/Abstract] OR "parkinson disease idiopathic"[Title/Abstract] OR "parkinson s disease"[Title/Abstract] OR "parkinson s disease idiopathic"[Title/Abstract] OR "parkinson s disease lewy body"[Title/Abstract] OR "primary parkinsonism"[Title/Abstract] OR "parkinsonism primary"[Title/Abstract] | 10-Nov-2025 | 152,739 |
|  | 2 | "parkinson disease"[Title/Abstract] OR "idiopathic parkinson disease"[Title/Abstract] OR "idiopathic parkinson s disease"[Title/Abstract] OR "lewy body parkinson disease"[Title/Abstract] OR "lewy body parkinson s disease"[Title/Abstract] OR "paralysis agitans"[Title/Abstract] OR "parkinson disease idiopathic"[Title/Abstract] OR "parkinson s disease"[Title/Abstract] OR "parkinson s disease idiopathic"[Title/Abstract] OR "parkinson s disease lewy body"[Title/Abstract] OR "primary parkinsonism"[Title/Abstract] OR "parkinsonism primary"[Title/Abstract] | 10-Nov-2025 | 139,399 |
|  | 1 | "Parkinson Disease"[MeSH Terms] | 10-Nov-2025 | 92,347 |
| Embase | #41 | #14 AND #23 AND #40 | 10-Nov-2025 | 202 |
|  | #40 | #26 OR #27 OR #28 OR #29 OR #30 OR #31 OR #33 OR #35 OR #36 OR #37 OR #38 OR #39 | 10-Nov-2025 | 1108319 |
|  | #39 | 'randomized experiment':ab,ti | 10-Nov-2025 | 749 |
|  | #38 | 'rct':ab,ti | 10-Nov-2025 | 78242 |
|  | #37 | 'random':ab,ti | 10-Nov-2025 | 534102 |
|  | #36 | 'randomized controlled trial':ab,ti | 10-Nov-2025 | 208972 |
|  | #35 | 'randomized controlled study':ab,ti | 10-Nov-2025 | 29636 |
|  | #34 | #16 AND #22 AND #24 AND #25 AND #32 | 10-Nov-2025 | 1322 |
|  | #33 | 'randomised controlled trial':ab,ti | 10-Nov-2025 | 54653 |
|  | #32 | ('surgery'/exp OR 'surgical technique'/exp OR 'surgical care':ti,ab,kw OR 'surgical correction':ti,ab,kw OR 'surgical operation':ti,ab,kw OR 'surgical practice':ti,ab,kw OR 'surgical repair':ti,ab,kw OR 'surgical research':ti,ab,kw OR 'surgical restoration':ti,ab,kw OR 'surgical service':ti,ab,kw OR 'surgical therapy':ti,ab,kw OR 'surgical treatment':ti,ab,kw) NOT ('non insulin dependent diabetes mellitus'/exp OR 'insulin resistance'/exp) NOT ('prediabetic state':ti,ab,kw OR 'type 2 diabetes mellitus':ti,ab,kw OR 'insulin':ti,ab,kw OR 'resistance':ti,ab,kw OR 'insulin resistance':ti,ab,kw OR 'sensitivity':ti,ab,kw OR 'insulin sensitivity':ti,ab,kw) | 10-Nov-2025 | 6802190 |
|  | #31 | 'randomised controlled study':ab,ti | 10-Nov-2025 | 4307 |
|  | #30 | 'controlled clinical trials, randomized':ab,ti | 10-Nov-2025 | 19 |
|  | #29 | 'trials, randomized clinical':ab,ti | 10-Nov-2025 | 31 |
|  | #28 | 'clinical trials, randomized':ab,ti | 10-Nov-2025 | 287 |
|  | #27 | 'randomized controlled trials as topic':ab,ti | 10-Nov-2025 | 8 |
|  | #26 | 'randomized controlled trial (topic)'/exp | 10-Nov-2025 | 303778 |
|  | #25 | 'surgical care':ti,ab OR 'surgical correction':ti,ab OR 'surgical operation':ti,ab OR 'surgical practice':ti,ab OR 'surgical procedures, operative':ti,ab OR 'surgical repair':ti,ab OR 'surgical research':ti,ab OR 'surgical restoration':ti,ab OR 'surgical service':ti,ab OR 'surgical therapy':ti,ab OR 'surgical treatment':ti,ab | 10-Nov-2025 | 356949 |
|  | #24 | 'preoperative period'/exp OR surgery*:ti,ab OR operation*:ti,ab OR 'operation care':ti,ab OR 'operative intervention':ti,ab OR 'operative surgical procedure':ti,ab OR 'operative treatment':ti,ab | 10-Nov-2025 | 3263983 |
|  | #23 | #15 OR #17 OR #18 OR #19 OR #20 OR #21 | 10-Nov-2025 | 186817 |
|  | #22 | 'fasting'/exp OR fasting:ti,ab OR starving:ti,ab OR drink*:ti,ab OR eat*:ti,ab OR meal:ti,ab OR meals:ti,ab OR feed:ti,ab OR solids:ti,ab OR intake*:ti,ab OR liquid*:ti,ab OR fluid*:ti,ab OR 'water'/exp OR npo:ti,ab OR 'nil per os':ti,ab OR 'nor per os':ti,ab OR 'diet restriction':ti,ab | 10-Nov-2025 | 3174288 |
|  | #21 | 'external rhythmic stimulation':ab,ti | 10-Nov-2025 | 10 |
|  | #20 | 'vibration':ab,ti | 10-Nov-2025 | 44565 |
|  | #19 | 'rhythmic auditory stimulation':ab,ti | 10-Nov-2025 | 310 |
|  | #18 | 'cue':ab,ti | 10-Nov-2025 | 44535 |
|  | #17 | 'cueing':ab,ti | 10-Nov-2025 | 5276 |
|  | #16 | 'blood glucose':ti,ab OR glucose:ti,ab OR blood:ti,ab OR 'glucose, blood':ti,ab | 10-Nov-2025 | 4069311 |
|  | #15 | 'cues':ab,ti | 10-Nov-2025 | 112885 |
|  | #14 | #1 OR #2 OR #3 OR #4 OR #5 OR #6 OR #7 OR #8 OR #9 OR #10 OR #11 OR #12 OR #13 | 10-Nov-2025 | 233927 |
|  | #13 | 'parkinsonism, primary':ab,ti | 10-Nov-2025 | 6 |
|  | #12 | 'primary parkinsonism':ab,ti | 10-Nov-2025 | 42 |
|  | #11 | 'parkinsons disease, lewy body':ab,ti | 10-Nov-2025 | 1 |
|  | #10 | 'parkinsons disease, idiopathic':ab,ti | 10-Nov-2025 | 1 |
|  | #9 | 'parkinsons disease':ab,ti | 10-Nov-2025 | 1334 |
|  | #8 | 'parkinson disease, idiopathic':ab,ti | 10-Nov-2025 | 5 |
|  | #7 | 'paralysis agitans':ab,ti | 10-Nov-2025 | 206 |
|  | #6 | 'lewy body parkinsons disease':ab,ti | 10-Nov-2025 | 0 |
|  | #5 | 'lewy body parkinson disease':ab,ti | 10-Nov-2025 | 5 |
|  | #4 | 'idiopathic parkinsons disease':ab,ti | 10-Nov-2025 | 44 |
|  | #3 | 'idiopathic parkinson disease':ab,ti | 10-Nov-2025 | 546 |
|  | #2 | 'parkinson disease':ab,ti | 10-Nov-2025 | 21204 |
|  | #1 | 'parkinson disease'/exp | 10-Nov-2025 | 231246 |
| Cochrane Library | #1 | MeSH descriptor: [Parkinson Disease] explode all trees | 11-Nov-2025 | 6417 |
|  | #2 | (Parkinson Disease):ti,ab,kw OR (Idiopathic Parkinson Disease):ti,ab,kw OR (Idiopathic Parkinson's Disease):ti,ab,kw OR (Lewy Body Parkinson Disease):ti,ab,kw OR (Lewy Body Parkinson's Disease):ti,ab,kw | 11-Nov-2025 | 14214 |
|  | #3 | (Paralysis Agitans):ti,ab,kw OR (Parkinson Disease, Idiopathic):ti,ab,kw OR (Parkinson's Disease):ti,ab,kw OR (Parkinson's Disease, Idiopathic):ti,ab,kw OR (Parkinson's Disease, Lewy Body):ti,ab,kw | 11-Nov-2025 | 14215 |
|  | #4 | (Primary Parkinsonism):ti,ab,kw OR (Parkinsonism, Primary):ti,ab,kw | 11-Nov-2025 | 418 |
|  | #5 | #1 or #2 or #3 or #4 | 11-Nov-2025 | 14357 |
|  | #6 | MeSH descriptor: [Cues] explode all trees | 11-Nov-2025 | 2529 |
|  | #7 | (Cues):ti,ab,kw OR (Cueing):ti,ab,kw OR (Cue):ti,ab,kw OR (rhythmic auditory stimulation):ti,ab,kw OR (Vibration):ti,ab,kw | 11-Nov-2025 | 13804 |
|  | #8 | (external rhythmic stimulation):ti,ab,kw | 11-Nov-2025 | 19 |
|  | #9 | #6 or #7 or #8 | 11-Nov-2025 | 13811 |
|  | #10 | MeSH descriptor: [Randomized Controlled Trials as Topic] explode all trees | 11-Nov-2025 | 62380 |
|  | #11 | (Randomized Controlled Trials as Topic):ti,ab,kw OR (Clinical Trials, Randomized):ti,ab,kw OR (Trials, Randomized Clinical):ti,ab,kw OR (Controlled Clinical Trials, Randomized):ti,ab,kw OR (randomised controlled study):ti,ab,kw | 11-Nov-2025 | 852357 |
|  | #12 | (randomised controlled trial):ti,ab,kw OR (randomized controlled study):ti,ab,kw OR (randomized controlled trial):ti,ab,kw OR (Random):ti,ab,kw OR (RCT):ti,ab,kw | 11-Nov-2025 | 980077 |
|  | #13 | (randomized experiment):ti,ab,kw | 11-Nov-2025 | 43517 |
|  | #14 | #10 or #11 or #12 or #13 | 11-Nov-2025 | 1008446 |
|  | #15 | #5 and #9 and #14 | 11-Nov-2025 | 220 |
| Web of Science | #1 | TS=(Parkinson Disease) OR TS=(Idiopathic Parkinson Disease) OR TS=(Idiopathic Parkinson's Disease) OR TS=(Lewy Body Parkinson Disease) OR TS=(Lewy Body Parkinson's Disease) OR TS=(Paralysis Agitans) OR TS=(Parkinson Disease, Idiopathic) OR TS=(Parkinson's Disease) OR TS=(Parkinson's Disease, Idiopathic) OR TS=(Parkinson's Disease, Lewy Body) OR TS=(Primary Parkinsonism) OR TS=(Parkinsonism, Primary) | 11-Nov-2025 | 165425 |
|  | #2 | TS=(Cues) OR TS=(Cueing) OR TS=(Cue) OR TS=(rhythmic auditory stimulation) OR TS=(Vibration) OR TS=(external rhythmic stimulation) | 11-Nov-2025 | 496671 |
|  | #3 | TS=(Randomized Controlled Trials as Topic) OR TS=(Clinical Trials, Randomized) OR TS=(Trials, Randomized Clinical) OR TS=(Controlled Clinical Trials, Randomized) OR TS=(randomised controlled study) OR TS=(randomised controlled trial) OR TS=(randomized controlled study) OR TS=(randomized controlled trial) OR TS=(Random) OR TS=(RCT) OR TS=(randomized experiment ) | 11-Nov-2025 | 1632426 |
|  | #4 | #1 AND #2 AND #3 | 11-Nov-2025 | 220 |
